# Supplementary material for: Distinct Responses to IL4 in Macrophages Mediated by JNK
Source: Cells. 2023 Apr 11;12(8):1127. doi: 10.3390/cells12081127 (PMC10136765; doi:10.3390/cells12081127)
Supplement: Supplementary file 1 [file cells-12-01127-s001.zip › cells-2244289-supplementary.pdf]

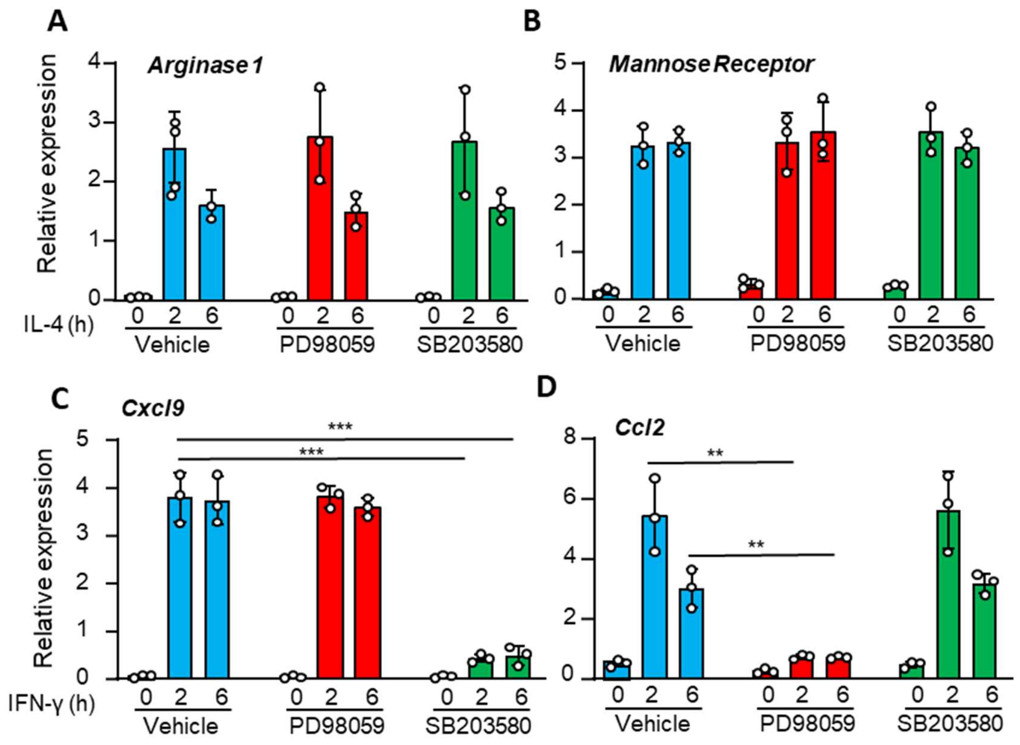

Supplementary Material Figure S1. Effects of MAPK inhibition on IL-4-mediated gene expression. A, Macrophages were preincubated for 1 h with inhibitors of MAPK signaling: PD98059 (50 μM; to block ERK activation), SB203580 (5 μM) (to inhibit p38), or vehicle (DMSO, as control). The cells were then stimulated for 2 or 6 h with IL-4. Control cells were left unstimulated. A and B *Arginase 1* and *Mannose-Receptor* genes that are IL4-JNK dependent (Figure 3), but ERK (PD98059 inhibitor) and p38 (SB203580) independent. C and D, Controls genes. *Cxcl9* is IL-4-p38 dependent and *Ccl2* is IL4-ERK dependent (19).

Supplementary Material Table S1. Antibodies: identification, source, application and dilution used.

Antibody	Identifier	Source	Application	Dilution
Rabbit polyclonal anti-Stat6	Cat# A11189; RRID: AB_2758452	Antibodies	IP	1:500
Mouse anti-Stat6 (Tyr 641)	Cat# 686002; RRID AB_2616820	Biolegend	Western blot and CHIP	1:1000
Goat anti-rabbit IgG (HRP)	Cat#A17345; RRID: AB_2772522	Antibodies	Western blot	1:2000
Goat anti-mouse HRP-conjugated IgG	Cat# ab6789; RRID: AB_955439	Abcam	Western blot	1:1000
Mouse anti β-actin IgG1	Cat# A5441; RRID: AB_476744	Sigma-Aldrich	Western blot	1:1000
Mouse anti-phosphoreine	Cat# ab7851; RRID: AB_306137	Abcam	Western blot	1:100
Rabbit anti-ERK1/ERK2	Cat# AF1576; RRID: AB_354872	R&D systems	Western blot	1:500
Rabbit anti-phospho-p38 (Thr180/Tyr182)	Cat# 9211; RRID: AB_331641	Cell Signalling	Western blot	1:1000
Rabbit polyclonal anti-JNK1	Cat# PA5-17889; RRID: AB_10982589	Invitrogen	Western blot	1:1000
Rabbit IgG anti-anti-KAT3B / p300	Cat# ab10485; RRID: AB_297224	Abcam	IP and CHIP	1:100
Rabbit IgG	Cat# 31887; RRID: AB_2532177	Invitrogen	Control CHIP	1:100
Mouse IgG	Cat# 14-4714-82; RRID:AB_470111	eBioscience	Control CHIP	1:100

Supplementary Material Table S2. Primer sequences for the various quantitative PCRs performed.

Transcript	Forward Primer	Reverse Primer
MKP-1	5’ GGACAACCACAAGGCAGACATC 3’	5’ GGCCTGGCAATGAACAAACA 3’
MKP-2	5’ TCGAAGACAACCACAAGGC 3’	5’ GGAAGCTGAAGACGAACTGC 3’
MKP-3	5’ TCGTCACACATCGAATCTGC 3’	5’ CAATGTCCGAGGAAGAGTCC 3’
MKP-4	5’ CCATCTCTGACCATTGGAGC 3’	5’ AGGCATCGTTGAGTGAGAGG 3’
MKP-5	5’ ACTTGATGAAGCACACACGG 3’	5’ TAAGGATTCTTGGCGTCACG 3’
MKP-7	5’ ATGGATGGTCCTTTGGCTAGG 3’	5’ CTCCTCACAATCAAGCCTGTCA 3’
PAC-1	5’ AGGCGGTTTCAAAAGCTTCC 3’	5’ GGTCAGAGTTGCTATTTTCGGC 3’
CPG21	5’ ATGAAGACCAAGCAGTTCCGG 3’	5’ ACTGAAGGAGCTGTCCCATGAA 3’
Arginase 1	5’ TTGCGAGACGTAGACCCTGG 3’	5’ CAAAGCTCAGGTGAATCGGC 3’
CCL22	5’ CTTCTGGACCTCAAAATCCTGC 3’	5’ TAGCTTCTTCACCCAGACCTGC 3’
CCL24	5’ TTGCTGCACGTCCTTTATTTCC 3’	5’ TGGCCCTTCTTGGTGATGAA 3’
IL-10	5’ CAAGGAGCATTTGAATTCCCTG 3’	5’ TGCTCCACTGCCTTGCTCTTA 3’
Mannose receptor	5’ GCTGAATCCCAGAAATTCCGC 3’	5’ ATCACAGGCATACAGGGTGAC 3’
CD163	5’ CTCTGTTCAGCTATTGGACGC 3’	5’ CGGAATTTCTGGGATTCAGCTT 3’
SOCS1	5’ GCATCCCTCTTAACCCGGTACT 3’	5’ ATAAGGCGCCCCCACTTAATG
$p21^{Waf1}$	5´ GAGCAAAGTGTGCCGTTGTCTC 3’	5´ TCAAAGTTCCACCGTTCTCGG 3’
c-myc	5’ AACAGCTTCGAAACTCTGGTGC 3’	5’ CGC ATCAGTTCTGTCAGAAGGA 3’
Hprt1	5’ ATCATTATGCCGAGGATTTGG 3’	5’ GCAAAGAACTTATAGCCCCC 3’
L14	5´ TCCCAGGCTGTTAACGCGGT.3’	5´ GCGCTGGCTGAATGCTCTG 3’
Sdha	5´ TGGGGAGTGCCGTGGTGTCA 3’	5´ CATGGCTGTGCCGTCCCCTG 3’
Cxcl9	5’ GATAAGGAATGCACGATGCTC 3’	5 TCTCCGTTCTTCAGTGTAGCAA 3’
Ccl2	5’ AGGTCCCTGTCATGCTTCTG 3´	5’ GCTGCTGGTGATCCTCTTGT 3’
